# Adjuvant stereotactic fractionated radiotherapy to the resection cavity in recurrent glioblastoma – the GlioCave study (NOA 17 – ARO 2016/3 – DKTK ROG trial)

**DOI:** 10.1186/s12885-017-3928-7

**Published:** 2018-01-03

**Authors:** Christoph Straube, Hagen Scherb, Jens Gempt, Jan Kirschke, Claus Zimmer, Friederike Schmidt-Graf, Bernhard Meyer, Stephanie E. Combs

**Affiliations:** 1 Klinik für RadioOnkologie und Strahlentherapie, Technische Universität München (TUM), Ismaninger Straße 22, 81675 Munich, Germany; 2 Institute of Computational Biology, Helmholtz Zentrum München, Deutsches Forschungszentrum fuer Gesundheit und Umwelt (GmbH), Ingolstaedter Landstr.1, 85764 Neuherberg, Germany; 3 Neurochirurgische Klinik und Poliklinik, Technische Universität München (TUM), Ismaninger Straße 22, 81675 Munich, Germany; 4 Abteilung für diagnostische und interventionelle Neuroradiologie, Technische Universität München (TUM), Ismaninger Straße 22, 81675 Munich, Germany; 5 Neurologische Klinik und Poliklinik, Technische Universität München (TUM), Ismaninger Straße 22, 81675 Munich, Germany; 6 Institut für Innovative Radiotherapie (iRT), Department of Radiation Sciences (DRS), Helmholtz Zentrum München, Ingolstädter Landstraße 1, 85764 Neuherberg, Germany; 7 Deutsches Konsortium für Translationale Krebsforschung (DKTK) - Partner Site Munich, 81675 Munich, Germany

**Keywords:** Glioblastoma, Recurrence, Re-irradiation, Gross total resection, Randomized trial, NOA, PFS

## Abstract

**Background:**

Glioblastoma relapses in the vast majority of cases within 1 year. Maximum safe resection of the recurrent glioblastoma can be offered in some cases. Re-irradiation has been established for the treatment of recurrent glioblastoma, too. In both cases, adjuvant treatment, mostly using temozolomide, can improve PFS and OS after these interventions. However, combining gross tumor resection and adjuvant re-radiotherapy to the resection cavity has not been tested so far.

**Methods/Design:**

In the multicenter two-armed randomized Phase II GlioCave Study, fractionated stereotactic radiotherapy to the resection cavity, after gross tumor resection of recurrent glioblastoma, will be compared to observation. Depending on the size of the target volume, a total dose of 46 Gy in 2 Gy per fraction or a total dose if 36 Gy in 3 Gy per fraction will be applied. Progression free survival will be the primary endpoint of the study.

**Discussion:**

Adjuvant treatment after gross tumor resection of recurrent glioblastoma is currently deemed to be limited to chemotherapy. However, re-irradiation has proven safety and tolerability in the treatment of macroscopic disease. Performing re-irradiation as an adjuvant measure after gross tumor resection has not been tested so far. The GlioCave Study will investigate the efficacy and the safety profile of this approach.

**Trial registration:**

The trial was prospectively registered at clinicaltrials.gov (NCT02715297, registration date February 29th, 2016). The protocol presented hereby refers to the version 1.2 of the protocol (January 11^th^, 2017).

## Background

Glioblastomas (GBM) refer to the most frequent and most aggressive primary brain tumors in adults; they are associated with a significant treatment resistance, in the primary situation as well as in the case of recurrence [1, 2]. Offering an extensive trimodal course of therapies, containing surgery, postoperative radiochemotherapy as well as adjuvant chemotherapy, survival still remains at a poor level. When first published from a randomized study in 2005, the radiochemotherapy regimen with temozolomide (TMZ) elevated median survival from 12.1 to 14.6 months [3]. However, despite extensive research, only minor progress has been achieved since almost 10 years [4–8].

In the vast majority of cases, GBM recurs within 1 year [3], and in most cases recurrence occurs locally [9]. Currently no standard of care can be defined for the treatment of relapsed GBM so far [10]. Thus, patients are treated within individual concepts, mostly based on retrospective studies or small, non-randomized trials [11].

Re-irradiation, especially when modern techniques such as radiosurgery (RS) or fractionated stereotactic radiotherapy (FSRT) has been established in the clinical routine and can be considered a safe and effective alternative for the treatment of recurrent glioblastoma [12–15]. Median overall and progression free survival ranges around 12 and 5 months, respectively, which is comparable to surgery [15, 16]. Generally, re-irradiation is applied in cases with macroscopic tumor remnants, not exceeding a maximum diameter of 4 cm; however, there is much controversy on the ideal target volume, the rationale for imaging during the treatment planning process, as well as to the ideal timepoint of re-irradition. In all cases surgery is evaluated in the case of recurrence, thus is must be discussed whether re-irradiation is only applicable in cases with tumor remnants. To overcome these limitations, it is worth considering multimodal concepts also for recurrent glioblastoma in order to achieve a prolongation of progression free survival.

As a first step, surgery is feasible especially if the tumor recurs in a not eloquent region, in patients with good physical performance status and if the recurrent tumor has a low tumor volume [17]. Furthermore, younger age might be a factor for better outcome [18], yet the prognostic value of second surgery is currently discussed controversially [19, 20].

Combinations of surgery and adjuvant systemic therapy as well as Re-irradiation with concurrent or adjuvant chemotherapy have been reported from several centers [21–24]. Especially the latter was able to achieve median overall survival of up 15 months, counting from the date of radiosurgery, in some series [16]. If only systemic therapy is possible in recurrent GBM due to its location, an early time point after former radiotherapy or the size, then it is associated with an overall survival of 6–8 month [11, 16].

Re-irradiation after surgery was reported to be superior to surgery alone in one prospective cohort, increasing OS from 13 weeks with surgery alone to 34 weeks with surgery plus chemotherapy or radiotherapy as adjuvant treatment [25]. Unfortunately, no target volumes were reported in these series. However, there is no data from randomized trials comparing observation after complete resection to an adjuvant treatment in the same situation. Bimodal local strategies combining complete resection followed by a second course radiotherapy have been reported in the context of brachytherapy, too. The median survival in several studies ranged from 52 weeks to 64 weeks after gross total resection with concurrent implantation of permanent ^125^Iodine seeds [26, 27]. No case of re-surgery for radionecrosis was reported in these two series, rendering adjuvant radiotherapy after GTR of a recurrent GBM as a safe treatment approach. Within a context of high dose rate brachytherapy, the GliaSite system was tested after maximal safe resection of recurrent glioblastoma in small series, gaining an overall survival of 9–13 months [19, 28]. Low dose rate as well as high dose rate brachytherapy are applied directly after surgery, thereby precluding sufficient MRI-based planning. Thus, residual tumor might have received only insufficient doses in this series. This would explain remarkably early progressive disease (16 weeks) described in Larson et al. in 2004 [26].

Within the present GlioCave study, we will investigate the impact of radiotherapy as an adjuvant treatment to patients that underwent gross tumor resection of a recurrent GBM.

## Methods/Design

### Study design

**Fig. 1:**
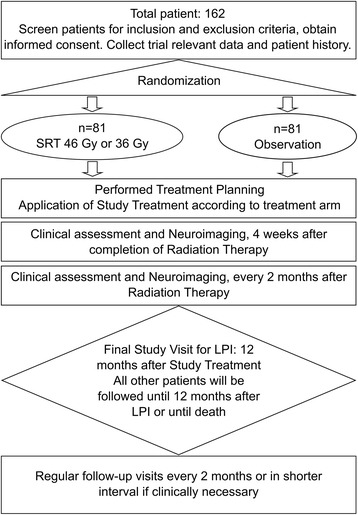
Flow chart of the GlioCave/NOA-17-Trial

GlioCave is a two-armed randomized multicentre open label phase II trial. Patients fulfilling the inclusion criteria will be 1:1 randomized into two arms (Fig. 1):

Arm A – Experimental Arm.

Postoperative stereotactic fractionated radiotherapy to a Total Dose of 46 Gy, 2 Gy single dose or 36 Gy in 3 Gy fractions depending in the size and location of the target volume.

Arm B – Standard Arm.

### Observation

Up to 24th April 2017, the study is active in two sides (Munich, Dresden). Activation of more sides is currently under preparation (Regensburg, Heidelberg, Cologne).

### Study objectives and endpoints

The trial is designed to allow the comparison of observation as a standard treatment to adjuvant radiotherapy after GTR of recurrent GBM.

The primary objective of the study is progression-free survival during the follow up phase of at least 12 months. Progression will be defined according to the RANO-HGG as well as to the MacDonald-Criteria [29, 30]. Progression free survival should be preferred as primary endpoint for trials on recurrent glioblastoma as the general aggressiveness of offered treatments influences overall survival in glioblastoma [31]. PFS is thus deemed to be less biased by further therapeutic approaches.

The secondary objectives are overall survival during the follow-up phase of at least 12 months (starting with diagnosis of recurrent disease). Toxicity will be assessed by type, incidence and severity according to the CTCAE v4.02. The EORTC QLQ-C30 version 3.0 questionnaire will be used to monitor for quality of life. Neurocognitive function will be tested at selected centers every 6 months, beginning after randomization. Patients will be followed until death. All study related data will be stored at the MiRO-Database of the Department of Radiation Oncology of the Technical University of Munich.

### Patients

Patients with the diagnosis of recurrent GBM presented will be evaluated and screened for the protocol. All patients fulfilling the inclusion and exclusion criteria will be informed about the study.

#### Inclusion criteria

- Unifocal, supratentorial recurrent glioblastoma
- Prior course of standard treatment
- Complete resection of all contrast enhancing areas
- age ≥ 18 years of age
- Karnofsky Performance Score ≥ 60%
- For women with childbearing potential, (and men) adequate contraception.
- Ability of subject to understand character and individual consequences of the clinical trial
- Written informed consent (must be available before enrolment in the trial)

#### Exclusion criteria

- Multifocal glioblastoma or gliomatosis cerebri
- Time interval of less than 6 months after primary radiotherapy
- Previous re-irradiation or prior radiosurgery of prior treatment with interstitial radioactive seeds
- refusal of the patients to take part in the study
- Patients who have not yet recovered from acute toxicities of prior therapies
- Known carcinoma < 5 years ago (excluding Carcinoma in situ of the cervix, basal cell carcinoma, squamous cell carcinoma of the skin) requiring immediate treatment interfering with study therapy
- Pregnant or lactating women
- Participation in another clinical study or observation period of competing trials, respectively.

### Radiotherapy

Treatment planning will be based on preoperative imaging studies as well as on postoperative MRI and planning MRI.

The clinical target volume (CTV) will contain the margins of the resection cavity of the recurrent tumor, including all contrast enhancing areas +5 mm.

A 1–3 mm expansion will be added to CTV to receive the planning target volume (PTV) depending on individual setup.

Radiotherapy will be prescribed to 95% of the PTV receiving the prescribed dose of either 46 Gy in 2 Gy per fraction or 36 Gy in 3 Gy per fraction.

### Systemic therapy

Chemotherapy is not part of this protocol. However, systemic treatments can be offered to the patients at best investigators choice.

### Statistics

The study is designed to demonstrate that addition of stereotactic fractionated radiotherapy to the resection cavity can significantly improve the progression free survival compared to a maximum safe resectionwithout a further adjuvant radiotherapy (standard treatment).

The data analytical and the statistical aspects of the study will be in accord with the Guidelines of International Conference on Harmonization (ICH):

- ICH E3: Structure and Contents of Clinical Study Reports
- ICH E6: Good Clinical Practice (GCP). Consolidated Guideline
- ICH E9: Note for Guidance on Statistical Principles in Clinical Trials

In the present context we will use an α = 0.1 (one-sided), as a one-sided type I error α = 0.1 presents little risk but increases the statistical power of the study. This is supported by a recent communication from the EORTC [32]. The sample size was calculated assuming a progression free survival (PFS) of 7 months after complete resection of a recurrent glioblastoma (control group) and a PFS of 10 months after additive radiotherapy to the resection cavity. With a planned total trial duration of 48 months, containing a recruitment phase of 36 months and a minimum follow up phase of 12 months and a hazard ration of 0.7, a sample of 81 patients per group is necessary to gain a statistical power of 0.8.

The primary endpoint will be analyzed on the per-protocol-group. Calculations will be made within the SAS-LIFETEST-Procedure. This includes non-parametric tests such as Kaplan-Meier-Estimators as well as lifetime-table-based calculations. Statistics for the secondary endpoint Overall Survival (OS) will be calculated similar.

Secondary endpoints will be described descriptively with the use of a Cox-regression model. Age, Karnofsky Performance Score, Recursive Partitioning Analysis, MGMT-status and initial IDH-1-status will be taken into account for the application of an ingression model.

### Interim analysis

The interim analysis for safety parameters will be done as soon as 20 patients have been treated and observed for at least 6 months.

### Ethical considerations

A positive vote from the local ethical committee of the technical university of Munich, Germany (continuous registration code 525/15 S) was obtained. The study was registered at chlinicaltrials.gov and received the ID NCT02715297.

The protocol received a positive vote from the “Unabhängiges Expertengremium der DEGRO”. By that, no further review is necessary.

### SPIRIT

The protocol was designed according to the Standard Protokocol Items: Recommendations for Interventional Trials (SPIRIT) criteria and underwent a peer review process.

## Discussion

Adjuvant radiotherapy is an established treatment in primary glioblastoma, independent to the extent of resection [3]. For a long time, a second course of radiotherapy was deemed to be unfeasible due to an expected increase in the risk of severe side effects in patients with completely resected recurrences. Adjuvant treatment after GTR of recurrent glioblastoma currently has therefore been limited to chemotherapy. However, during the last decade, re-irradiation-approaches for macroscopic disease have been established successfully with only limited toxicity [11, 15]. With a small margin around the resection cavity, an acceptable amount of brain tissue will undergo re-irradiation (Manuscript under preparation, Straube et al.). It is therefore worth considering to offer a second course of adjuvant radiotherapy to patients with GTR.

GlioCave is the first phase II trial that will investigate the efficacy as well as the toxicity-profile of this approach.

## Abbreviations

CT: Computed tomographie
CTV: Clinical target volume
DEGRO: Deutsche Gesellschaft für Radioonkologie
FSRT: Fractionated stereotactic radiotherapy
GBM: Glioblastoma
GCP: Good clinical practice
GTR: Gross tumor resection
GTV: Gross tumor volume
ICH: International Conference of Harmonization
KPS: Karnofsky performance score
MRI: Magnet resonance imaging
OS: Overall survival
PFS: Progression free survival
PTV: Planning target volume
RS: Radiosurger
TMZ: Temozolomide
